# Intrapleural instillation of autologous blood for persistent air leak in spontaneous pneumothorax- is it as effective as it is safe?

**DOI:** 10.1186/1749-8090-5-61

**Published:** 2010-08-17

**Authors:** Dimos Karangelis, Georgios I Tagarakis, Marios Daskalopoulos, Georgios Skoumis, Nicholaos Desimonas, Vasileios Saleptsis, Theocharis Koufakis, Athanasios Drakos, Dimitrios Papadopoulos, Nikolaos B Tsilimingas

**Affiliations:** 1 Department of Cardiovascular and Thoracic Surgery, University Hospital of Thessaly, Larissa, Greece

## Abstract

**Objective:**

The aim of the present study was to evaluate the efficacy of autologous blood pleurodesis in the management of persistent air leak in spontaneous pneumothorax.

**Patients and methods:**

A number of 15 patients (10 male and 5 female) were included in this prospective study between March 2005 and December 2009. The duration of the air leak exceeded 7 days in all patients. The application of blood pleurodesis was used as the last preoperative conservative method of treatment in 12 patients. One patient refused surgery and two were ineligible for operation due to their comorbidities. A blood sample of 50 ml was obtained from the patient's femoral vein and immediately introduced into the chest tube.

**Results:**

A success rate of 27% was observed having the air leak sealed in 4 patients in less than 24 hours.

**Conclusion:**

Despite our disappointingly poor outcome, the authors believe that the procedure's safety, convenience and low cost establish it as a worth trying method of conservative treatment for patients with the aforementioned pathology for whom no other alternative than surgery would be a choice.

## Introduction

Persistent air leak is frequently encountered in thoracic surgery especially after pulmonary surgery or pneumothorax. It prolongs patient's hospital stay and is considered to be a difficult problem regarding its management [1,2]. Pleurodesis is an excellent method used to treat air leak and it is feasible by means of surgery, autologous blood and several intra-pleural chemical agents such as talc powder, tetracycline, doxycycline, bleomycine ect. Regardless of the method, surgical or conservative, the goal of pleurodesis is to provoke adhesions between the parietal and the visceral pleura and thus minimize the space between the two layers. In the case of surgery this is achieved by mechanical irritation of the parietal pleura while sclerotic agents induce dense adhesions chemically. Autologous blood irritates the pleural surfaces and is considered to act by formatting a patch of clotted blood (fibrin), which can potentially adhere to the lung parenchyma that produces the leak. Nevertheless, according to some authors blood can also act like a sclerotic agent causing a few adhesions [3]. Although much has been written about the different types of pleurodesis, no specific guidelines have been determined, a fact indicative of the lack of consensus of experts in the subject. The aim of this article was to discuss our department's five-year experience with autologous blood pleurodesis for the management of prolonged air leak after spontaneous pneumothorax.

## Patients and methods

### The participants

Our study was conducted between March 2005 and December 2009 and comprised 15 patients with persistent air leak resulting from their first episode of spontaneous pneumothorax. Exclusion criteria were the presence of bullous disease confirmed with a CT scan before the induction of pleurodesis and the positive anamnesis of spontaneous pneumothorax, as the latter would constitute a clear indication for surgical intervention. The patients were in majority young, with age range 16 to 72 years and a mean age of 31.9 years. In 12 of them the blood pleurodesis was applied as the last conservative method of treatment prior to surgery while one of them refused operation and 2 suffered from pulmonary fibrosis and severe chronic obstructive disease respectively (table 1). Given the advanced lung disease in the last 2 cases, (FEV1 < 1.5 lt) both we and the patients were reluctant to consider surgical pleurodesis. In those two patients the blood pleurodesis was repeated two days after the first attempt (table 1). The air leak was defined as persistent when it exceeded seven days in accordance with the definition of air leakage proposed in literature [4,5]. The "blood patch" was introduced after this time limit of seven days. All patients with spontaneous pneumothorax and air leakage exceeding seven days were included in the study. In total, 15 out of 142 potentially study eligible patients met the final time related criteria and were recruited for the protocol. The patients were informed and a written copy of their consent was obtained prior to the procedure.

**Table 1:** Patients' data and results

	Patients with blood pleurodesis efficacy (Group A)	Patients submitted to surgery (Group B)	Patients ineligible for surgery (Group C)	Patients total	Comparisons	Statistical Significance
Size sample (male)	4 (3)	9 (5)	2 (2)	15 (10)	-	-
Mean age (years)	37.5	20.77	71	31.9	A vs. B p = 0.09	NS
					A vs. C p = 0.01	SS
					B vs. C p < 0.01	SS
Mean hospitalization (days)	12	19.66	13.5	16.8	A vs. B p = 0.01	SS
					A vs. C p = 1.0	NS
					B vs. C p = 0.1	NS
Attempts of pleurodesis	1	1	2	-	-	-
Success rate	4/15 (27%)	9 out of 9 (100%)	2/2 discharged with Heimlich valve	-	A vs. B p < 0.01	SS

The table shows basic data of the three groups placed in a comparative form. The statistical analysis was based on the one way ANOVA and the χ^2 ^criterion methods. The level of statistical significance was set at a level for p < 0.05. From the analysis following conclusions can be drawn.

1. As far as mean age concerned there was no significant difference between the group A and group B patients p = 0.09; on the contrary there were statistically significant differences between the patients of groups A vs. C and B vs. C (p = 0.01 and p < 0.01 respectively). This comparison was based on the one way ANOVA method.

2. Regarding the mean hospitalization duration we concluded to a statistically significant difference between the patients of the groups A vs. B (p = 0.01). No such differences were noted in the comparison between groups A vs. C and B vs. C (p = 1.0 and 0.1 respectively). This comparison was based on the one way ANOVA method.

3. In concern to the success rate of the different procedures it is obvious from the statistical comparisons that surgical treatment clearly supervenes the pleurodesis approach p < 0.01. This comparison was based on the χ^2 ^criterion.

### The technique

A blood sample of 50 ml was gained from the patient's femoral vein and immediately introduced into the chest tube without using anticoagulants. We used a standard chest tube of 28F and large syringes of 18 gauge and 0.9 mm. After the application, the chest tube was not clamped but raised over the patient's level in order to prevent blood running backwards in the drainage. Clamping of the chest tube during and after instillation of blood into the pleural cavity was avoided in order to prevent recurrence or deterioration of pneumothorax [6]. Patients were instructed to receive different positions in bed (left decubitus, right decubitus, Trendelenberg and Fowler), every 30 minutes for 2-3 hours, in order to achieve ideal distribution of blood in the thoracic cavity. The procedure was carried out at bedside under aseptic conditions. No sedative or analgesic was administered. The next day a chest x-ray was carried out. In case of air leak sealing we had the chest tube removed the same day. None of the patients presented difficulty in breathing, cough or other side effects during the procedure and we did not observe significant decrease in the value of haematocrit.

## Results

Blood pleurodesis succeeded in 4 out of 15 patients (27%) with the air leaks sealing within 24 hours of blood injection in all cases (table 1). Two patients were discharged with a Heimlich valve after 2 unsuccessful attempts of pleurodesis, while 8 patients with persistent air leak on the 3^rd ^or 4^th ^day after the application of pleurodesis were finally submitted to surgery. One patient developed pneumonia 48 h after the procedure and was treated with oral antibiotics. More specifically we administered amoxicillin and clarythromycin per os thus having the pneumonia resolved in 12 days. He underwent the operation after the retreat of his disease. All 9 patients who underwent surgery were submitted to open thoracotomy. After the lung pathology was treated (stapling of blebs and apicectomy), the parietal pleura was irritated by mechanical abrasion with a sponge soaked in dextrose 35% water solution. We had 100% seal of the air leak after the operation (table 1). In a 3-month follow up with a plain chest x-ray no recurrence of pneumothorax was demonstrated in the 13 patients, one patient died due to his comorbidities and one remained with a Heimlich valve free of other symptoms.

## Discussion

It was Robinson who first introduced blood pleurodesis for chronic spontaneous pneumothorax, followed by Dumire some years later, who applied it for persistent pulmonary air leak [7,8]. Although fever, pleural effusion and empyema have been reported with this method [1,2], there are several other reports that accent it as the safest method of pleurodesis in persistent air leak after pulmonary surgery and spontaneous pneumothorax [4,9-12]. Numerous other sclerotic agents have been used to produce pleural symphysis with different advantages and drawbacks. From tetracycline and doxycycline, to quinacrine, bleomycin, talc, interferon or even silver nitrate [13-16]. Despite the fact that many surgeons seek the best method of pleurodesis among these sclerotic agents, we consider blood pleurodesis as the safest and most preferable intervention in our case, especially because our sample of patients consisted mostly of minor ages. Our primary goal was to avoid the possible toxic side effects of chemical agents especially in our young patients [3]. We did not have the expected results, although the technique we followed was adhered to the recommendations of literature. We performed the procedure after seven days of air leak which is the optimal time according to many authors [2,8,9] and we used big sized chest tubes and syringes to avoid catheter obstruction. The volume of blood is a controversial point among different authors. Many perform the pleurodesis by instillation of 50 ml of blood [2,5,12], while others use 120 ml or 150 ml [4,10] thus introducing a completely different approach. We preferred the injection of 50 ml only once (with the exception of multi-morbid patients) because minimal exposure of the patients to all infection risks resulting from tubing manipulations was our major concern. In those two ineligible for surgery we considered it appropriate to repeat the method 48 hours after the first attempt, before reaching the final decision for a Heimlich valve. In regard to the other patients, it was their minor mean age that dictated the less aggressive strategy we followed (one attempt). In similar case series success rates vary between 75% [5] and 84% [1]. As concerning our success percentage of 27%, we believe that it could possibly be significantly higher should we had applied the method more than once.

Two major points we can comment on from our analysis as it can be clearly extracted from table 1 are: i) the mean age of the patients that were not submitted to surgery was significantly higher than the mean age from the operated ones. This is an expected result considering the multiple comorbidities and risks associated with an increased age. ii) The operated patients had a prolonged hospitalization period compared to the rest of the patients. These findings clearly show that blood pleurodesis, when successful, decreases hospital stay and can be the method of choice for patients not amendable to surgical interventions.

## Conclusion

Although our results were not encouraging enough regarding the success rate, we still consider blood pleurodesis to be a worth trying method for treating pneumothorax with persistent air leak. It is safe, painless, well tolerated and low cost technique that can be performed even by clinicians who lack great experience, as long as strict attention to aseptic conditions is paid. Also it is a rather useful option of conservative treatment when dealing with patients of high morbidity and mortality surgical risks who are ineligible for surgery. Conclusively blood pleurodesis is an "only-win" method as there are minimal complications related to it, while the few patients in whom it succeeds enjoy great benefits.

## Competing interests

The authors declare that they have no competing interests.

## Authors' contributions

DK made a thorough literature research, and was the chief author in terms of building the paper. GT co-authored the paper in terms of major contribution. MD performed literature research. GS and ND gave their specialist advice on scientific issues of the paper. VS checked the paper. TK assisted with the linguistics and performed literature research. AD and DP performed literature research. NT checked the final version of the manuscript. All authors read and approved the final manuscript. The manuscript is not under consideration and has not been published by another journal.
